# Modulation of early and late event-related potentials by emotion

**DOI:** 10.3389/fnint.2012.00102

**Published:** 2012-11-08

**Authors:** Sarah J. Hart, Nathaniel Lucena, Katherine M. Cleary, Aysenil Belger, Franc C. L. Donkers

**Affiliations:** ^1^Department of Psychiatry, University of North Carolina at Chapel Hill School of MedicineChapel Hill, NC, USA; ^2^Department of Psychology, Tilburg UniversityTilburg, Netherlands

**Keywords:** contingent negative variation, emotion, anticipation, distraction

## Abstract

Although emotionally salient stimuli influence higher order information processing, the relative vulnerability of specific stages of cognitive processing to modulation by emotional input remains elusive. To test the temporal dynamics of emotional interference during executive function, we recorded event-related potentials (ERPs) while participants performed an effortful anticipation task with aversive emotional and neutral distracters. Participants were presented with a modified delayed Stroop task that dissociated the anticipation of an easier or more difficult task (instructional cues to attend to word vs. color) from the response to the Stroop stimulus, while aversive and neutral pictures were displayed during the delay period. Our results indicated a relative decrease in the amplitude of the contingent negative variation (CNV) during aversive trials that was greater during the early anticipatory phase than during the later response preparation phase, and greater during (the more difficult) color than word trials. During the initial stage of cue processing, there was also significant interaction between emotion and anticipatory difficulty on N1 amplitude, where emotional stimuli led to significantly enhanced negativity during color cues relative to word cues. These results suggest that earlier processes of orientation and effortful anticipation may reflect executive engagement that is influenced by emotional interference while later phases of response preparation may be modulated by emotional interference regardless of anticipatory difficulty.

## Introduction

The presence of emotionally salient stimuli is known to influence how the brain processes information. Depending upon the specific timing in which emotional and cognitive systems are engaged, input from emotional circuitry can enhance or disrupt neural activity related to higher order executive function. While presentation of negative emotionally salient distracters subsequent to the onset of an executive task can lead to disruption of sustained attention processes (Arnsten and Goldman-Rakic, 1998; Dolcos and McCarthy, 2006), several studies have shown that emotional stimuli presented prior to or concurrent with cognitive processing can also lead to an improvement in performance (Gray et al., 2002; Schupp et al., 2007). Several studies by our group and others have characterized the dynamics of these opposing systems with functional neuroimaging (Blair et al., 2007; Hart et al., 2010). While these studies inform about the global effects of emotional interference, most executive functions rely on a sequence of complex operations, including stimulus selection, rule encoding, anticipation and response preparation, as well as response selection, and execution. By investigating how emotional interference may modulate these specific stages of processing, we may gain a better understanding of the underlying mechanisms of emotional-executive interactions.

Understanding the effects of emotional interference during specific stages of executive function is critical for not only understanding how affective interference modulates cognition in healthy brain development and function, but also for understanding the affective and cognitive dysregulation in a number of brain disorders, including schizophrenia, post-traumatic stress disorder (PTSD), affective disorders, and autism. Dysregulation of these executive-emotional interactions may trigger the onset or exacerbation of cognitive deficits associated with emotional-executive regulation mechanisms. In order to gain a more complete understanding of emotion-cognitive interactions in both the healthy brain and in psychiatric disorders, it is important to investigate how emotion influences cognition at different temporal stages of processing, as well as how it influences cognition in specific neural circuits. Therefore, in the current study we aimed to further elucidate the mechanisms underlying executive-emotional interactions by examining the influence of emotional distracters across different temporal stages of executive processing by using event-related potentials (ERPs). We aimed to investigate how specific stages of cognitive processing, such as the anticipation of cognitive effort and the preparation for a motor response, may differ in their vulnerability to emotional modulation.

The ability to prepare for an upcoming task is a critical aspect of executive function and involves recruitment of several neural circuits across multiple stages of processing (Brass and von Cramon, 2004). Electroencephalographic (EEG) recordings have long been used to study the different processes involved in the coordination of goal-directed behavior by means of slow brain potentials. The best known examples in this regard are the readiness potential (RP), the stimulus preceding negativity (SPN), and the contingent negative variation (CNV). The RP (Kornhuber and Deecke, 1965) reflects the timing of a future voluntary movement, the SPN (Brunia, 1988; Leynes et al., 1998) reflects anticipatory attention for an upcoming stimulus and the CNV (Walter et al., 1964) reflects the preparation of a signaled movement and the simultaneous anticipatory attention for the imperative stimulus. When a longer period (3–5 s) between a warning/instructional stimulus and an imperative/probe stimulus is used, the CNV can be divided into early and late phases (Brunia and van Boxtel, 2001). The early phase of the CNV primarily reflects orientation and anticipatory cognitive control processes in response to a warning stimulus, and the late phase of the CNV primarily (but not necessarily) reflects preparation for an upcoming motor response (Brunia and van Boxtel, 2001). Many studies have demonstrated that the CNV amplitude is increased during attentional demand and is significantly reduced when a distracting stimulus is present (Teece, 1972; Gontier et al., 2007). The neural generators for the CNV have been reported to include the prefrontal cortex (PFC), anterior cingulate cortex (ACC), premotor cortex and supplementary motor area for the early phase (Gomez et al., 2004; Lutcke et al., 2009), and basal ganglia, prefrontal, pre-motor, and dorsal ACC (Ikeda et al., 1997; Gomez et al., 2003; Lutcke et al., 2009) for the late phase.

Several previous studies have examined the interactions between anticipatory processing and emotion but have primarily focused on the role of cognitive strategies during anticipation of an upcoming task-relevant emotional stimulus, reflecting the modulation of attentional resource allocation. Moser et al. (2009) recently demonstrated that preparation for cognitive reappraisal of an impending negative emotional stimulus modulates the SPN. Greater engagement of cognitive resources during anticipatory processes may therefore suppress the effect of subsequent negative emotional stimuli, as the process of mental preparation for the impending emotional stimulus allows the brain to regulate the degree of emotional processing through increased prefrontal cortical activity (Goldin et al., 2008). Anticipatory processes are also modulated by preparation for emotionally salient inputs, with enhanced amplitudes during anticipation of positive emotional stimuli (Casement et al., 2008).

While these studies have demonstrated that anticipatory processes can influence task-relevant emotional processing, there is a relative lack of knowledge about the opposite effect, that is, how task-*irrelevant* emotional interference may affect anticipatory processes for an upcoming (difficult) cognitive task. Because the direction of attention allocation may influence how emotional and anticipatory processes interact, it is important to understand how each process may impact the other. By using a slow wave ERP paradigm we aimed to address the effect of emotion on anticipatory processing by measuring how the different temporal phases of preparation for goal-directed behavior may be modulated by emotional interference. We chose to use emotional stimuli with negative valence based on a multitude of previous findings showing disruption of prefrontal activity (Dolcos and McCarthy, 2006; Arnsten, 2009; Qin et al., 2009). To test the temporal dynamics of emotional interference, we used a delayed Stroop task that dissociates the instructional cue from the response in order to separately examine anticipatory processes and the executive response. The Stroop task allows for the measurement of cognitive control processes required to suppress an automatic response (reading a word) while identifying the color in which the word is printed. When these stimulus attributes are incongruent with one another (e.g., the word “RED” in green print), it is more difficult to suppress the prepotent response to read the word. In order to assess anticipatory difficulty, we first presented an instructional cue indicating whether the subject should subsequently identify the presented word (the easier reading response) or the print color (requiring more cognitive control). A delay period followed, during which emotional distracter images were presented while participants prepared to respond to an upcoming Stroop stimulus. The delayed Stroop task design allowed for the dissociation of separable cognitive phases so that the effects of emotional interference could be assessed over time. Additionally, the task was previously validated with fMRI (MacDonald et al., 2000) and therefore had the advantage of allowing for inference of the neural circuits likely involved.

We focused our analyses on the ERP components elicited by the presentation of the cue (N1) and during the anticipatory period across the delay (CNV) prior to the executive response to the Stroop stimulus. By presenting emotional stimuli during the delay period, we measured the modulation across the early (cognitive control) and late (motor preparatory) phases of the CNV, and tested how anticipation of increased task demand (with presumed increased PFC engagement) affected this emotional modulation. Furthermore, analysis of the N1 component allowed us to determine whether emotional interference also disrupts attentional processing at the earliest sensory phase, prior to the emergence of the CNV. The N1 has previously been found to be the earliest component modulated by emotional stimuli in a passive viewing task (Foti et al., 2009) and has been associated with increased vigilance for emotionally threatening stimuli (Shackman et al., 2011).

Given previous findings that the PFC is involved in effortful anticipation (MacDonald et al., 2000) and may be disrupted by negative emotional interference (Dolcos and McCarthy, 2006), we predicted that ERP components elicited during the early preparatory task phase would show greater emotional modulation under conditions of increased anticipatory effort than ERP components elicited during the later preparatory phase of the Stroop task. We also expected that participants would be slower to respond during more effortful anticipatory trials, with further decline in performance during aversive emotional interference.

## Materials and methods

### Participants

We collected EEG data from 12 healthy participants between the ages of 19–34 (mean age = 22.9), including six males and six females. All participants were right-handed, with no current or past history of substance abuse or neurologic/neuropsychiatric disorders, and reported normal or corrected-to-normal vision. Participants gave informed consent as approved by the UNC Institutional Review Board.

### Experimental procedure

Continuous EEG data was recorded during a modified Stroop task designed to temporally dissociate effortful anticipatory processing from the implementation of cognitive control. The classic Stroop task requires participants to respond to a color word (e.g., “RED”) by either reading the word or responding to the ink color in which the word is written. The ink color may be congruent (e.g., “RED” in red ink) or incongruent (e.g., “RED” in blue ink), requiring additional cognitive control to suppress the prepotent reading response. To assess the anticipatory phase, we first presented an instructional cue indicating whether the subject should identify the presented word (the prepotent reading response) or the print color (requiring more cognitive control). Following a delay period during which the participants prepared to respond, the Stroop stimulus (probe) was presented. Three possible congruent probes (the word “RED” in red, the word “BLUE” in blue, and the word “GREEN” in green) and six possible incongruent probes (the word “RED” in blue, the word “RED” in green, the word “BLUE” in red, the word “BLUE” in green, the word “GREEN” in red, and the word “GREEN” in blue) were used. The Stroop probe stimuli were randomly varied to be congruent and incongruent (each comprising 50% of the total trials). Participants were directed to respond to the probe as quickly and accurately as possible pressing one out of three buttons. The buttons 7, 8, and 9 on a standard USB keyboard were mapped according to the three colors possible (i.e., red = 7, blue = 8, and green = 9). Participants always responded with their index (button 7), middle (button 8) or ring finger (button 9) to red, blue, or green colors, respectively. The inter-trial interval was randomized between 3 and 6 s at 250 ms intervals. Additionally, prior to beginning the task, all participants performed a practice session with 20 total trials using scrambled images in place of the International Affective Picture System (IAPS) distracter images. This session allowed participants to become familiarized with the button response mappings, and to learn that identification of the word was easier than the print color, without introducing any habituation to the emotional images.

In order to modulate emotional processing, each trial included presentation of a task-irrelevant picture from the IAPS database (Lang et al., 2005). This database includes images that have standardized ratings for arousal and valence on a scale of 1–9, with higher numbers indicating greater arousal and more positive emotional valence. Within the experiment, 50% of the trials were aversive (valence mean = 2.57, *SD* = 0.81; arousal mean = 6.46, *SD* = 0.46) and 50% were neutral (valence mean = 5.27, *SD* = 0.8; arousal mean = 3.27, *SD* = 0.62). The images used in the neutral condition had significantly higher valence [*F*_(1, 158)_ = 444.34, *p* < 0.0001] and lower arousal ratings [*F*_(1, 158)_ = 1340.83, *p* < 0.0001] than the aversive condition.

**Figure 1:**
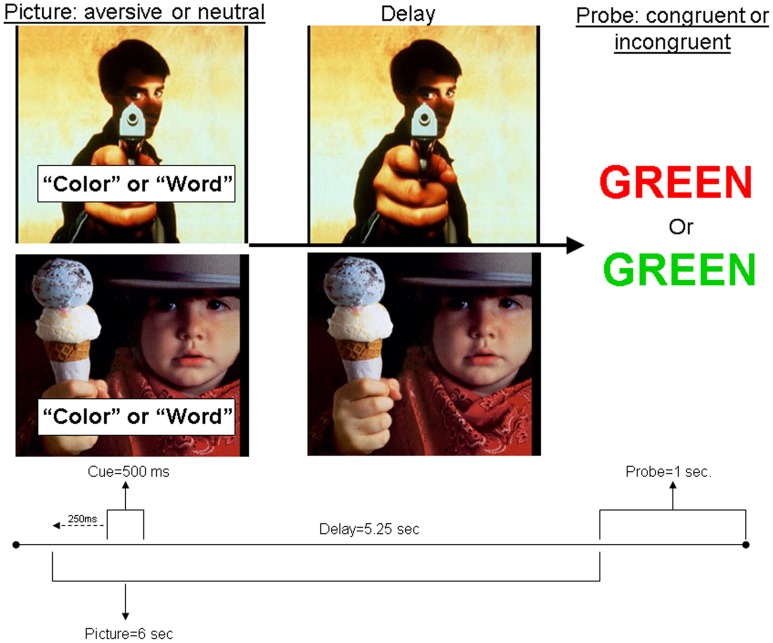
**Schematic of task design**.

Each trial began with the presentation of an aversive or neutral IAPS distracter image (Figure 1). Beginning at 250 ms after the onset of the trial, a cue was imposed over the IAPS distracter image for 500 ms displaying either “word,” instructing the participant to respond to the probe by reading the printed word, or “color,” instructing the participant to respond to the probe using the color of the text. Each cue type was randomly presented in 50% of the total trials across the experiment. After the cue, there was a 5250 ms delay during which the IAPS distracter image remained on the screen. Following the delay, the IAPS image disappeared and a Stroop probe was displayed for 1 s. There were 160 total trials (each with a unique image) presented across 8 runs, with 20 trials per run.

#### Electrophysiological recording

Participants were seated comfortably in a sound-attenuated, dimly lit booth. Pictures were displayed at a distance of about 80 cm at eye level on a 19′ Dell flat panel monitor. Stimulus presentation was controlled by Presentation software, version 12.1 (Neurobehavioral Systems, Albany, CA). Continuous EEG data was collected using an elastic cap containing 32 electrodes at frontal (F7, F3, Fz, F4, F8), frontocentral (FT7, FC3, FCz, FC4, FT8), central (T7, C3, Cz, C4, T8), centroparietal (TP7, CP3, CPz, CP4, TP8), and occipital (O1, Oz, O2) scalp locations. The right mastoid served as the reference electrode and AFz as the ground. Bipolar recordings of the vertical and horizontal electro-oculogram (EOG) were obtained by electrodes placed above and below the right eye and on the outer canthus of each eye, respectively. EEG and EOG data were sampled at a rate of 500 Hz and bandpass filtered online between 0.05 and 100 Hz, with a narrow 60 Hz notch filter used to reduce main power frequency interference. Continuous data was collected and analyzed off-line using a NeuroScan 4.4 system (Neurosoft, Inc., Sterling, VA).

### Data processing

Response latencies and percentage of correct responses were calculated for each subject. All incorrect trials or trials containing responses less than 200 ms and greater than 1000 ms from onset of the probe were excluded from further analyses. Continuous EEG data was filtered offline with a 30 Hz (24 dB/octave) low-pass filter and visually inspected for movement artifacts, and incorrect behavioral responses were removed from the analyses. EEG data sets from each participant were corrected for eye-movements using regression analysis as implemented in Neuroscan Edit 4.4 (Semlitsch et al., 1986).

For analysis of the CNV, continuous data was divided into 8 s long epochs, spanning from 500 ms before the distracter stimulus to 7500 ms thereafter and including a 1500 ms post-probe period. All epochs were baseline corrected using the first 200 ms of the pre-distracter interval, with all conditions using the same averaged baseline. Any epoch containing amplitudes exceeding ±100μV at any electrode was excluded from further analyses. All remaining epochs were averaged together based on stimulus category.

In order to separately analyze the early and late components of the CNV, average amplitudes were computed for two time windows during the CNV delay period: 250–2250 ms following the cue onset (early CNV) and 3250–5250 ms following cue onset (late CNV). Based on prior findings of the scalp distribution for the two phases of the CNV (Zappoli et al., 2000; Gomez et al., 2001, 2003), the early phase was analyzed at frontal sites (F3, Fz, and F4) and the late CNV at parietal sites (P3, Pz, and P4), with both phases analyzed at electrode Cz. To analyze the response related to the “word” or “color” instructional cue (N1), negative peak amplitude and latency was analyzed from a window between the onset of the cue and 250 ms following cue onset at electrodes Fz and Cz. These electrodes were chosen in order to visualize the experimental effects on the N1 and CNV at the same site. Additionally, we performed the N1 analyses at occipital sites, including O1, Oz, and O2. For statistical analysis, a series of repeated measures ANOVAs were performed with electrode location, cue type and emotion as within-subjects factors. We note that while a P3 component was generated following the Stroop probe stimulus, the analysis of this component was outside the scope of our hypotheses, which focused specifically on emotional modulation of anticipatory processing. Because of our sample size limitation, and because our hypotheses were focused on the role of cue and emotion on behavior and ERP measures, we chose not to include a Three-Way ANOVA integrating the probe condition in our analyses.

## Results

### Behavioral data

**Figure 2:**
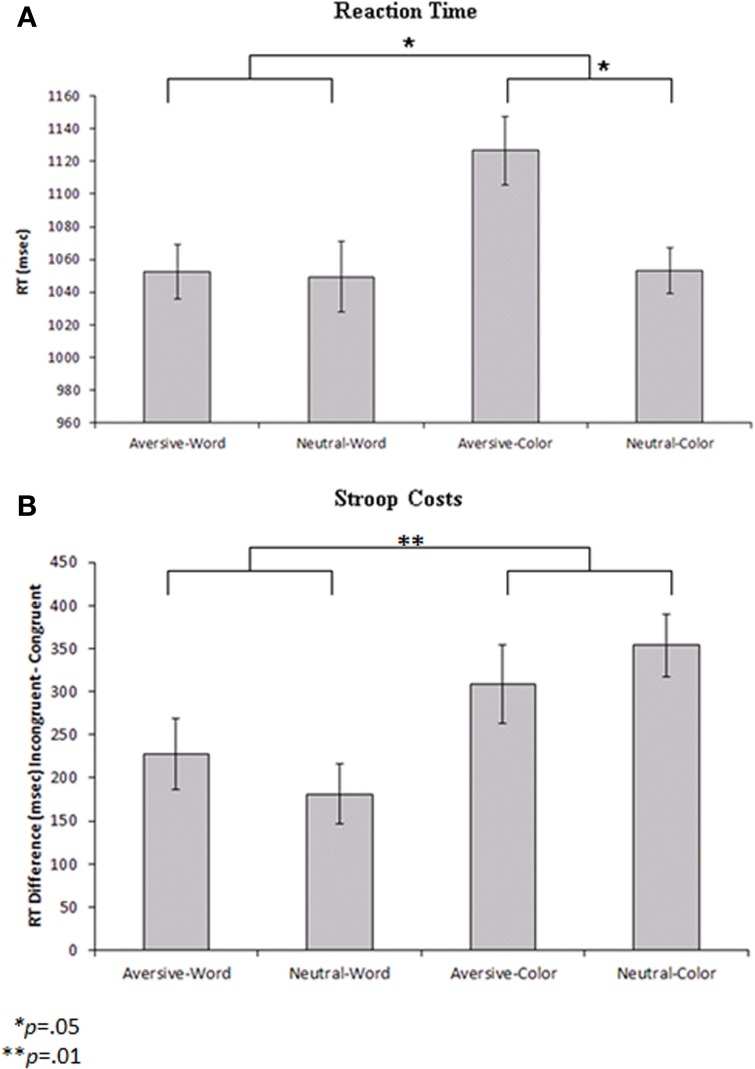
**(A)** Reaction times to the probe stimuli for each condition with repeated-measures error bars. Color cues led to significantly slower reaction times than word cues (*p* = 0.05), and aversive trials led to slower reaction times than neutral trials within the color condition (*p* = 0.05). **(B)** Reaction time differences between incongruent and congruent probe stimuli (Stroop costs) for each condition with repeated-measures error bars. Color cues led to significantly greater Stroop costs than word cues (*p* = 0.01).

The behavioral findings for accuracy and RT for all conditions are presented in Table 1. An ANOVA was performed using RT on correct trials as the dependent variable. Color trials were found to be associated with significantly slower RTs relative to word trials [*F*_(1,11)_ = 4.98, *p* = 0.05]. Additionally, aversive emotional trials were associated with significantly slower RTs compared to neutral trials [*F*_(1, 11)_ = 4.75, *p* = 0.05]. A significant Cue by Emotion interaction [*F*_(1, 11)_ = 7.7, *p* = 0.02] indicated that the slowing of RTs during color trials was primarily driven by the emotional interference (Figure 2A). *Post-hoc* tests indicated that aversive pictures led to significantly slower RT than neutral pictures on color trials [*F*_(1, 11)_ = 13.95, *p* = 0.003], but no significant effect of emotion was found on word trials. Additional analyses of accuracy revealed no significant main effects of cue or emotionality, and no significant cue by emotionality interaction effect.

**Table 1:** **Behavioral Results**.

**Congruent**				**Incongruent**			
	**Aversive**	**Neutral**	**Average**		**Aversive**	**Neutral**	**Average**
**Accuracy (% correct)**							
Color: Mean, SD	89.81 (4.82)	94.09 (3.18)	91.95 (4.55)	Color: Mean, SD	87.01 (10.62)	83.1 (9.13)	85.06 (10.62)
Word: Mean, SD	90.05 (4.95)	93.85 (3.24)	91.95 (4.53)	Word: Mean, SD	87.21 (6.04)	77.7 (3.6)	82.46 (6.88)
Average	89.93 (4.78)	93.97 (3.14)	91.95 (4.49)	Average	87.11 (8.45)	80.4 (7.33)	83.76 (8.53)
**Reaction time (ms)**							
Color: Mean, SD	1001.89 (232.66)	914.59 (272.85)	958.24 (251.96)	Color: Mean, SD	1310.97 (354.2)	1268.18 (313.5)	1289.57 (327.85)
Word: Mean, SD	931.39 (223.39)	961.6 (305.64)	946.5 (262.26)	Word: Mean, SD	1158.47 (322.35)	1142.87 (342.4)	1150.67 (325.31)
Average	966.64 (225.95)	938.1(284.36)	952.37 (254.48)	Average	1234.72 (340.24)	1205.52 (327.37)	1220.12 (330.62)

Additional exploratory analyses were performed to determine how the cue and emotionality conditions affected Stroop probe processing. For the Stroop probe stimuli, incongruent trials were associated with lower percent accuracy [*F*_(1, 11)_ = 26.64, *p* < 0.001] and slower RTs [*F*_(1, 11)_ = 63.79, *p* < 0.001] than congruent trials. To analyze the behavioral effects of cue type and emotionality on the Stroop effect, a repeated measures ANOVA was performed with Cue (word vs. color) and Emotion (aversive vs. neutral) entered as within subjects factors and Stroop cost (RT difference between incongruent and congruent trials) used as the dependent variable. The results indicated that there were significantly greater Stroop costs on color trials (mean difference = 331.33 ms) than word trials (mean difference = 204.17 ms) [*F*_(1, 11)_ = 9.81, *p* = 0.01] (Figure 2B). *Post-hoc* analyses indicated that color trials were associated with slower RTs than word trials, both during the incongruent [*F*_(1, 11)_ = 17.95, *p* = 0.001] and congruent conditions [*F*_(1, 11)_ = 6.18, *p* = 0.03]. There was no significant effect of emotionality and no Emotion by Cue interaction on Stroop costs.

### N1 measures

**Figure 3:**
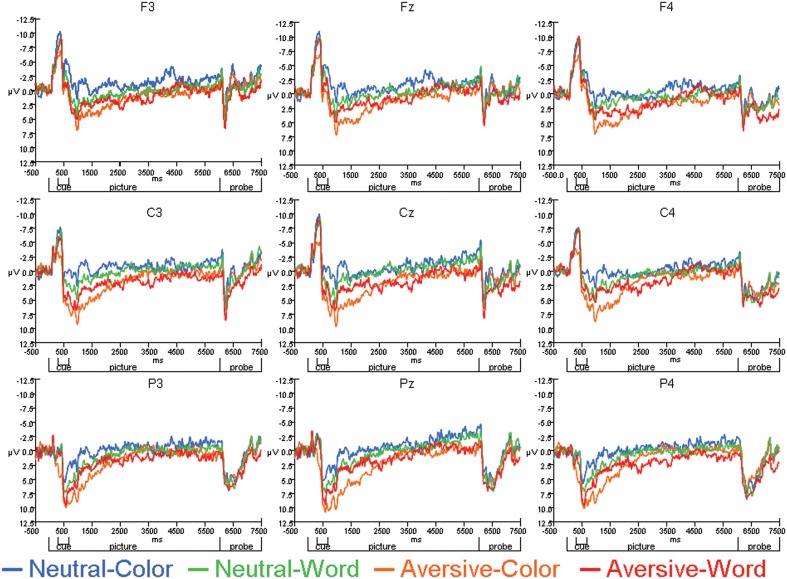
**Average ERPs for Neutral-Color, Neutral-Word, Aversive-Color, and Aversive-Word conditions across individual electrode sites**.

**Figure 4:**
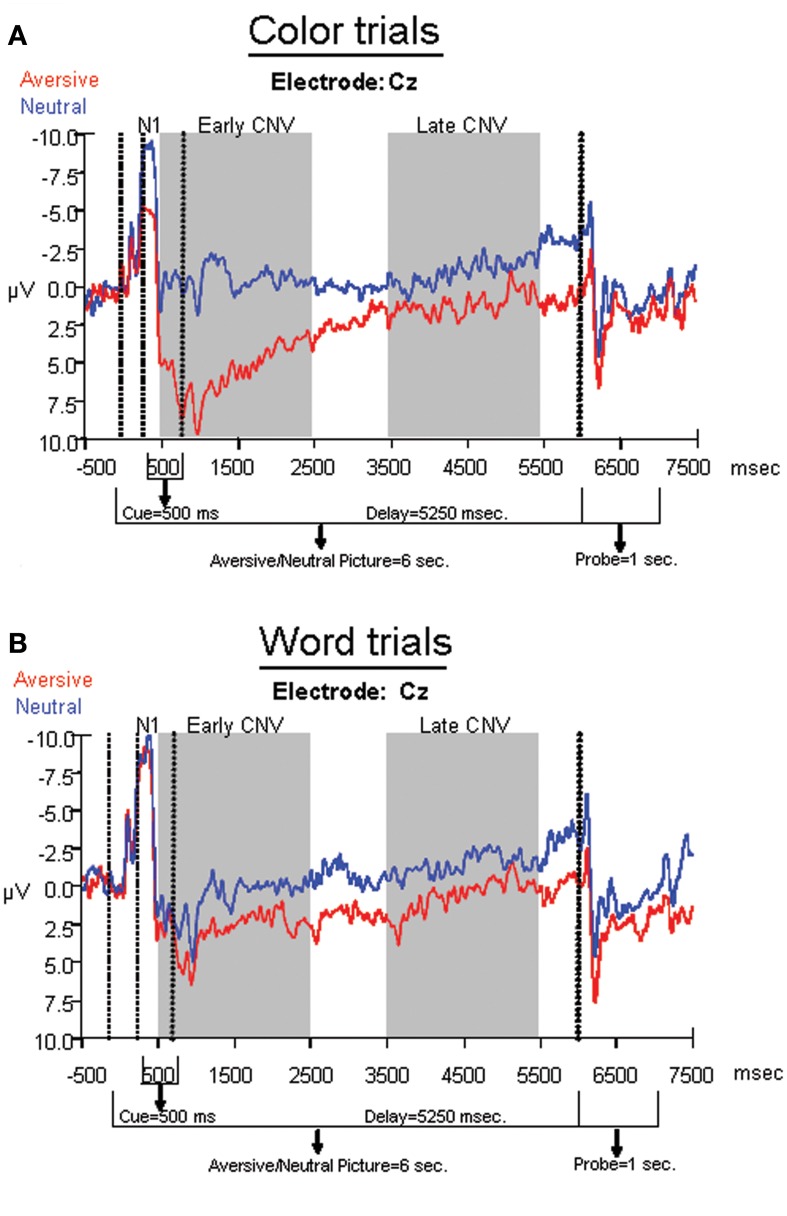
**Average ERPs for aversive and neutral conditions within (A) color trials and (B) word trials.** Electrode Cz was chosen for display purposes, as it was analyzed across the N1, early, and late CNV components.

The average ERPs across the entire trial for the neutral-word, neutral-color, aversive-word, and aversive-color conditions are presented from an array of nine electrodes in Figure 3. Analyses at electrodes Fz and Cz indicated that aversive trials led to significantly decreased negativity in N1 amplitude relative to neutral trials [*F*_(1, 22)_ = 9.59, *p* = 0.005], but no significant effect of Cue was found. A significant Cue by Emotion interaction was found [*F*_(1, 22)_ = 14.91, *p* < 0.001], where the relative decrease in N1 amplitude by emotion was significant on color trials [*F*_(1, 22)_ = 19.47, *p* < 0.001] (Figure 4A), but not word trials (Figure 4B). No significant effect of electrode location was found. Analyses at occipital electrodes showed similar effects, with N1 amplitude significantly decreased during aversive trials [*F*_(1, 33)_ = 11.26, *p* = 0.002] and a significant Cue by Emotion interaction [*F*_(1, 33)_ = 11.79, *p* = 0.002]. Analyses of N1 peak latency indicated that word cues showed a trend for slightly faster peak latency than color cues [*F*_(1, 22)_ = 3.88, *p* = 0.06], but no effects of Emotion, Emotion by Cue interaction, or electrode location were found.

### Early and late CNV measures

A repeated measures ANOVA was performed on frontal sites (F3, Fz, and F4) to determine effects of Emotion and Cue on the early CNV average amplitude. The ANOVA revealed that emotional trials led to significantly decreased negativity relative to neutral trials [*F*_(1, 33)_ = 20.66, *p* < 0.001], but no significant effect of Cue was found. A significant Emotion by Cue interaction [*F*_(1, 33)_ = 12.06, *p* = 0.002] revealed that the early CNV average amplitude was significantly modulated by Emotion during the processing of the color trials [*F*_(1, 33)_ = 24.72, *p* < 0.001], but not the word trials^1^ (Figures 3, 4). Additionally, no significant effects of electrode location were found.

Another repeated measures ANOVA was performed on parietal sites (P3, Pz, and P4) to determine effects of emotion and cue on late CNV average amplitude. The ANOVA revealed that aversive trials led to significantly decreased negativity compared to neutral trials [*F*_(1, 33)_ = 5.96, *p* = 0.02], but no significant main effect of Cue was found. Additionally, no significant Cue by Emotion interaction was found, nor was there a significant effect of electrode location.

## Discussion

The overall goal of this study was to examine the temporal characteristics of emotional interference and modulation of executive anticipatory processes. Our results indicated that N1 and early CNV amplitudes during early orienting and anticipatory processing were significantly reduced in the presence of emotional interference, but only when preparation for greater cognitive control was required. These results were consistent with the reaction time data, indicating that the process of anticipating a cognitively demanding task is more vulnerable to disruption from emotionally salient information than a less demanding task. In contrast, for the late CNV component associated with preparation for a motor response, aversive images led to a significant reduction in negative going amplitude regardless of anticipatory effort. These results suggest that while the N1 and early CNV may provide markers of executive engagement that are sensitive to emotional modulation, motor preparatory processes indexed by the late CNV are affected in general by emotional interference but are not differentially engaged by anticipatory difficulty. Emotional input may therefore lead to disruption of activity across the entire phase of anticipatory processing, but the engagement of additional cognitive control mechanisms leads to additional modulation by emotional interference. These findings reveal differential effects of emotional interference on specific sequential operations of executive processing and suggest potential implications for psychiatric disorders that are characterized by dysfunction in these mechanisms. Indeed, individual differences in salience attribution to emotional cues, or increased attention allocation due to “perceived” task complexity may alter early response preparation and anticipation during higher order executive tasks.

As the interpretation of ERPs is limited by poor spatial resolution, the integration of complementary measures that identify the underlying neural circuits will allow for improved characterization of how emotional interference affects anticipatory processing. Because our task was modeled on the delayed Stroop paradigm used in the fMRI study by MacDonald et al. (2000), it allows us to infer the specific neural circuits that may be recruited by the task while gaining temporal information from ERP recording. MacDonald et al. (2000) reported a double dissociation in that anticipation of a more difficult task (color trials) recruited the dorsolateral PFC (dlPFC) to a greater degree, while response conflict at the Stroop probe was associated with greater anterior cingulate activity. These regions were interpreted to potentially comprise a feedback loop in which top-down control (dlPFC) and evaluative processes (ACC) interact to maintain optimal task performance. Based on these findings, the results of the current study suggest that the emotional modulation we observed in the ERP data was likely related to disruption of the engagement of the PFC early in the trial during effortful anticipation. Although the anterior cingulate has been implicated as a source for the early CNV (Gomez et al., 2004) and is engaged during anticipation and performance monitoring in general (MacDonald et al., 2000), the previous fMRI findings suggest that it was likely not engaged to a greater degree during the more difficult anticipation condition in our task until the Stroop probe stimulus occurred.

The CNV was found in the current study to be modulated in an interactive way by both attentional demand and emotional interference. It has been classically described as reflecting the amount of attention allocated to an impending subsequent stimulus, which requires a response from the subject (Teece, 1972). Many additional studies have demonstrated that the CNV amplitude is increased during attentional demand, and is significantly reduced when a distracting stimulus is present (Teece, 1972; Gontier et al., 2007). The CNV has also been found to be sensitive to arousal effects. A study using similar emotional stimuli as the current design demonstrated modulation of the CNV in individuals with PTSD, with greater arousal to negative IAPS pictures associated with larger CNV amplitudes (Wessa and Flor, 2007). Increased CNV amplitudes have also been found after administration of amphetamine, and decreased amplitude following administration of barbiturates, which lead to enhanced or suppressed arousal, respectively (Kopell et al., 1974). As behavioral performance is also improved by pharmacologically-induced arousal, the concomitant CNV amplitude changes have been interpreted to reflect increased ability to attend to task-relevant features and resist distraction (Teece, 1972; Kopell et al., 1974). The current study's findings differ in that task-irrelevant emotionally arousing stimuli led to behavioral interference and decreased CNV amplitude on cognitively demanding trials. Our results reflect the role of emotional arousal as a distracter that likely inhibits the prefrontal-mediated processes underlying the CNV (as in Dolcos and McCarthy, 2006), but modulation of the task relevance or timing in which emotionally arousing stimuli are presented may differentially affect CNV amplitude and performance.

The timing of the emotional and executive requirements of the task may be particularly critical in determining how these processes interact over time. The current study's findings are consistent with the idea that more difficult cognitive processing may be impaired by emotional interference due to competition for resources (Pessoa, 2009). Therefore, the earlier phase of anticipatory processing, likely reflecting greater engagement of PFC, may also reflect relatively increased competition for attention from emotional stimuli. However, a previous study from our group showed that when emotional interference is presented immediately prior to the engagement of a Stroop task, the need for greater executive function overrides the emotional attenuation effect (Hart et al., 2010). Executive function engagement is therefore likely to be modulated differently depending on the relative timing of emotional inputs and task goals, which may determine whether emotion has an enhancing or impairing impact on cognition. The current study's results suggest that emotional interference during anticipatory processing has an impairing effect during specific early phases of processing, when prefrontal mechanisms are likely more engaged.

Several limitations should be considered when interpreting the current study. Our study design lacked sufficient power to allow for the assessment of how the ERP response to the Stroop probe conditions (congruent vs. incongruent) interacted with cue difficulty and emotionality of the pictures. While our behavioral data indicated that Stroop costs were affected by cue difficulty but not emotional interference, it is possible that increased engagement of the ACC during response to conflicting Stroop probes could be differentially modulated by prior emotional distraction and increased preparatory difficulty. Additionally, our study design did not allow us to make a direct comparison between emotional effects on the early and late phases of the CNV. By manipulating the interval between the emotional stimuli and the subsequent Stroop probe, it would be possible to directly investigate the effects of the onset of emotional stimuli on different phases of the trial. A third limitation of the current study is the possibility of habituation effects to the emotional pictures, over the course of a single trial or the experiment as a whole. Within-trial habituation effects could potentially differentially influence the early and late phases of the CNV. However, our results showed that the aversive condition still led to a decrease in the late CNV during the less-demanding word trials, with the decrease being more pronounced on more difficult color trials. These results are consistent with findings indicating that differential emotional modulation of other ERP components are resistant to time-on-task effects (Olofsson and Polich, 2007). Finally, our design did not distinguish between positive and negative emotional modulation of the CNV. The relationship between positive emotion and distractibility is unclear, but a recent study showed that the CNV was not modulated by induced positive mood prior to a cognitive control task (van Wouwe et al., 2011). Future studies that directly compare positive and negative distracters during the CNV delay interval may address the potential differing roles of stimulus valence in CNV modulation.

An additional consideration is that the current study's design cannot directly address whether the emotional interference effect could have been specific to color processing compared to semantic processing, as opposed to attributing it to task difficulty. While we believe that participants did likely anticipate a more difficult task during color trials as a result of the training period prior to the study, our design cannot determine whether there may be some specificity about anticipation of color processing that would be more vulnerable to emotional interference. However, given that prior findings have shown PFC engagement in the same task (MacDonald et al., 2000) and as emotional interference has been found to disrupt the PFC (Dolcos and McCarthy, 2006), we believe that our results suggest that greater need for cognitive control likely accounted for the interactions between emotion and cue.

The current findings may be particularly relevant for several psychiatric disorders that are characterized by changes in prefrontal-limbic interactions, such as schizophrenia, autism, and PTSD. Recent studies have reported alterations in CNV amplitudes in control subjects but not schizophrenia patients in response to sensory cue processing (Dias et al., 2011; Bickel et al., 2012) as well as anticipation of upcoming target events (Ford et al., 2010). Given these observations, it is important to elucidate factors that may contribute to a reduced early CNV attenuation. The current data suggest that emotional context differentially affects the early phases of the CNV, and suggest that disorders where prefrontal pathophysiology has been implicated may lead to greater susceptibility to emotional interference, particularly during earlier phases of the CNV generation and response preparation when executive and attentional mechanisms are more engaged. The future use of a similar study design that allows for temporal dissociation between phases of processing may be helpful for directly investigating potential patterns of neural dysfunction in executive-emotional interactions that characterize these disorders.

Overall, the current study's findings help to elucidate the temporal dynamics of emotional-executive interactions by showing that emotional distracters can be shown to elicit differential effects depending on the phase of processing. By segregating different phases of neural activity involved in anticipatory processing, emotional distracters can be found to selectively depress both early orienting and anticipatory processing only under conditions where cognitive control mechanisms are more engaged. The later phase of motor preparatory activity, in contrast, may be more generally affected by emotional interference without being dependent on increased preparatory activity for a more difficult task. It may be that the later phase engages prefrontal and premotor mechanisms that are functionally affected by emotional input, but are not engaged by greater need for executive control. In contrast, the need for greater executive engagement at an earlier stage of processing may exacerbate the effect of emotion. Future studies that directly manipulate task difficulty within a sensory domain may rule out whether the current study's effects are specific to differences between color and word processing. Further studies may focus on combining these approaches with fMRI to better localize the neural sources of the dynamic changes in cognitive-emotional interactions, and manipulating the context in which emotional stimuli may interfere with or potentiate executive functions.

### Conflict of interest statement

The authors declare that the research was conducted in the absence of any commercial or financial relationships that could be construed as a potential conflict of interest.

## Footnotes

^1^What we describe in this section as a relative “decrease in negativity” could also be described as a relative “increase in positivity.” However, even if we follow the latter line of reasoning—in that the aversive figure triggers a large positive component in the color condition but fails to do so in the word condition—we would still reach the same conclusion. That is: early orienting and effortful anticipation are significantly reduced in the presence of emotional interference, but only when preparation for greater cognitive control is required.
